# A Novel Mitochondrial-Related Gene Signature for the Tumor Immune Microenvironment Evaluation and Prognosis Prediction in Lung Adenocarcinoma

**DOI:** 10.1155/2022/5366185

**Published:** 2022-05-25

**Authors:** Yin-ping Li, Gui-xia Liu, Zhan-ling Wu, Ping-hua Tu, Guang Wei, Man Yuan, Min-hua Zhong, Ke-lan Deng

**Affiliations:** Department of Respiratory and Critical Care Medicine, The Center Hospital of Xiaogan, Xiaogan, Hubei, China

## Abstract

Lung adenocarcinoma (LUAD) remains the most common deadly disease and has a poor prognosis. More and more studies have reported that mitochondrial-related genes (MTRGs) were associated with the clinical outcomes of multiple tumors solely. In this study, we aimed to develop a novel prognostic model based on MTRGs. Differentially expressed MTRGs were identified from TCGA-LUAD and GSE31210 cohorts. Univariate Cox regression analysis was utilized to screen differentially expressed MTRGs that were related to prognosis of LUAD. Then, LASSO Cox regression analysis was used to develop a prognostic signature. ESTIMATE was used for estimating the fractions of immune cell types. In this study, we identified 44 overlapping differentially expressed MTRGs in TCGA-LUAD and GSE31210 cohorts. Among 44 overlapping differentially expressed MTRGs, nine genes were associated with prognosis of LUAD. When the penalty parameter lambda was the minimum, there were six genes meeting the conditions of constructing the signature, including SERPINB5, CCNB1, FGR MAOB, SH3BP5, and CYP24A1. The survival analysis suggested that prognosis of patients in the high-risk group was significantly worse than that in the low-risk group. Cox regression analyses showed that the risk score was an independent predictor of LUAD prognosis. As with the results of ESTIMATE score, the degree of immune cell infiltration in the low-risk group was higher than that in the high-risk group, such as TIL, Treg, and B cells. In addition, TMB and cancer stem cell infiltration were higher in the low-risk group than the high-risk group. In conclusion, we developed a novel MTRG signature acting as a negative independent prognostic factor. In the future, individualized treatments and medical decision-making may benefit from using the predicted model.

## 1. Introduction

Lung adenocarcinoma (LUAD) is the most common form of lung cancer and the leading cause of cancer-related death in the United States and around the world [1]. As if that was not bad enough, LUAD's incidence and death are increasing [2]. Surgery, chemotherapy, targeted therapy, and immunotherapy are among the therapeutic options for LUAD. Despite these options, the 5-year survival rate for people with LUAD varies from 4 to 17 percent, depending on the condition and treatment options [3]. Histopathological diagnosis and the tumor staging system are still the primary sources of the prognostic information in cancer treatments [4, 5]. Traditional methods, on the other hand, are inadequate for accurately assessing the results of LUAD patients [6]. Therefore, the development of robust and reliable prognostic biomarkers is essential to assist clinicians in optimizing therapy approaches.

The use of microarray technology in conjunction with bioinformatics tools has recently been demonstrated to be effective in the identification of novel genes associated with cancer progression, diagnosis, and prognosis [7, 8]. Thus, bioinformatics analysis is a viable and valuable tool for screening differently expressed genes (DEGs) from microarray data and identifying the essential genes associated with LUAD development and prognosis [9, 10]. Clinical and molecular biomarkers for LUAD have recently been examined in various researches in an effort for better management and therapy of this disease. In predicting the prognosis of LUAD, comprehensive genetic profiling has found common genetic changes, such as family with sequence similarity 83 (FAM83) members, the zinc finger homeobox 3 (ZFHX3) mutation, and epidermal growth factor receptor (EGFR) mutation [11–13]. And Bruton's tyrosine kinase (BTK), lncRNA JPX, and serum heparin-binding growth factor (HDGF) were found to be associated with poor mortality in LUAD patients by transcriptome profiling [14–16]. In addition, signatures based on multiple gene expressions, such as glycolysis-related genes, autophagy-related lncRNAs, and tumor microenvironment-related genes, have been demonstrated to display a strong ability in predicting the clinical outcome of LUAD patients [17–19]. A multigene biomarker, as opposed to a single molecular marker, was more accurate and sensitive in its prediction powers.

Mitochondria, the cell's “powerhouse,” are involved in a wide range of cellular processes, such as cell growth and death, signaling transduction, and energy metabolism [20, 21]. Nuclear mitochondrial-related DNA/RNA and mitochondrial DNA/RNA were discovered by next-generation sequencing in mitochondrial disorders [22]. Nonstructural nuclear mitochondrial-related genes (MTRGs), such as COA6, COA5, and NDUFAF1, were related to cardioencephalomyopathy; besides, structural nuclear MTRGs, such as DUFV2, NDUFB10, and NDUFS2, were related to cardiomyopathy [23–26]. Other investigations have conclusively shown that mitochondrial malfunction is linked to the development of cancers [27, 28]. In this study, we aimed to develop a novel prognostic model based on MTRGs.

## 2. Materials and Methods

### 2.1. Microarray Data and Gene Collection

A total of 594 TCGA-LUAD mRNA files, corresponding clinical information, and mutation data were obtained from TCGA database (https://cancergenome.nih.gov/) in February 2022. Of the 594 samples, 535 were tumor tissues and 59 were normal tissues. The GSE31210 cohort, which was consisted of 226 LUAD tissues and 20 normal tissues, was downloaded from the GEO database (https://www.ncbi.nlm.nih.gov/geo/). TCGA-LUAD and GSE31210 cohorts were used to screen differentially expressed genes. In addition, GSE72094 (including 442 LUAD patients), GSE3141 (111 lung cancer patients), and GSE50081 (181 lung cancer patients) were downloaded from the GEO database as verification cohorts. A total of 1513 mitochondrial-related genes (MTRGs) were collected from GSEA and previous studies [29, 30].

### 2.2. Screening of Differentially Expressed MTRGs

After removing samples that lack follow-up time and survival status, there were 490 samples in TCGA-LUAD cohort. Differentially expressed MTRGs were identified from TCGA-LUAD and GSE31210 cohorts, respectively, with FDR < 0.05 and ∣logFC | >1. Overlapping differentially expressed MTRGs between two cohorts were used for subsequent analyses. To visualize the expression of overlapping differentially expressed MTRGs, a heat map was produced using the “pheatmap” package [31]. To exhibit the connection between these genes, a correlogram was drawn using the “corrplot” package [32].

### 2.3. Development and Verification of Prediction Signature

Univariate Cox regression analysis was utilized to screen differentially expressed MTRGs that were related to prognosis of LUAD. Then, LASSO Cox regression analysis was used to develop a prognostic signature using the “glmnet” package. The parameter lambda of LASSO was determined using10-fold cross-validation. The signature was established as follows: risk score = sum (each gene′s expression × corresponding coefficient). Patients were assigned to high-risk (HR) and low-risk (LR) groups based on the median score. Survival curves were drawn using “survminer” and “survival” packages, and corresponding K-M curves were produced. To evaluate the ability of the signature to distinguish patients at different risks, PCA and tSNE analyses were performed using the “Rtsne” package [33]. In addition, univariate and multivariate Cox regression analyses were conducted to determine whether the signature was independent of clinical characteristics.

### 2.4. Subgroup Analysis of Clinical Characteristics

To confirm the correlation between signature and clinical features, a subgroup analysis was performed using TCGA-LUAD cohort. According to available clinical data, patients were divided into several subgroups, including age (≤65 and >65 groups), gender (male and female groups), tumor grade (I-II and III-IV groups), T stage (T1-2 and T3-4 groups), N stage (N0-1 and N2-3 groups), and M stage (M0 and M1 groups).

### 2.5. Immunity Analysis

ESTIMATE algorithm, which calculates the proportion of stromal and immune cells in cancer samples based on gene expression data, was conducted. Single sample gene set enrichment analysis (ssGSEA) was used to quantify the enrichment level of immune functions and infiltration degrees of immune cells. What is more, the relationship between immune cell infiltration and gene expression was obtained from the TIMER database (https://cistrome.shinyapps.io/timer/).

Immune checkpoint blockade (ICB) therapy has achieved unprecedented advances in cancer treatment. To assess whether there were differences in ICB therapy among patients in different risk groups, we analyzed the relationship between risk score and the expression of immune checkpoints. The Tumor Immune Dysfunction and Exclusion (TIDE, http://tide.dfci.harvard.edu/) website records the immunotherapy response of patients with NSCLC and provides TIDE scores on anti-PD-1 and anti-CTLA-4 responses. Higher tumor TIDE prediction score is associated with poor efficacy of ICB therapy. What is more, it provided the dysfunction score of T cells.

### 2.6. Tumor Mutation Burden Analysis

Tumor mutation burden (TMB) represents the number of tumor-derived new antigens and is a key determinant of ICB response. Therefore, we analyzed the TMB of each sample in TCGA-LUAD cohort and compared the mutation level between two groups. Then, according to the combination of mutation level and risk score, patients were divided into four groups for survival analysis.

### 2.7. Cancer Stem Cell Infiltration Analysis

Cancer stem cell index represents the infiltration degree of cancer stem cell. To observe the difference of stem cell infiltration between risk groups, the cancer stem cell index was calculated at the DNA and RNA levels, respectively.

### 2.8. Gene Set Enrichment Analysis

Gene set enrichment analysis (GSEA), including GO and KEGG enrichment analyses, was used to determine whether there were enrichment differences between the gene sets of the two risk groups in phenotypic categories.

### 2.9. Exploring Sensitive Drugs

CellMiner (http://discover.nci.nih.gov/cellminer) includes NCI-60 data. The drug sensitivity information file was downloaded from it. Drugs that were not marked with FDA approval were excluded. The results of the first 16 analyses were visualized according to the *p* value from small to large row.

### 2.10. Statistical Analysis

Besides online analysis, all statistics were conducted using R Project (Version 4.1.2). Kaplan-Meier curves were plotted, and a log-rank test was used to check the significant difference in OS between groups. Univariate and multivariate Cox proportional hazard regression analysis was also performed to access the association between risk score and OS. All results were considered statistically significant with *p* value less than 0.05.

## 3. Results

### 3.1. Identification of Differentially Expressed MTRGs

**Figure 1:**
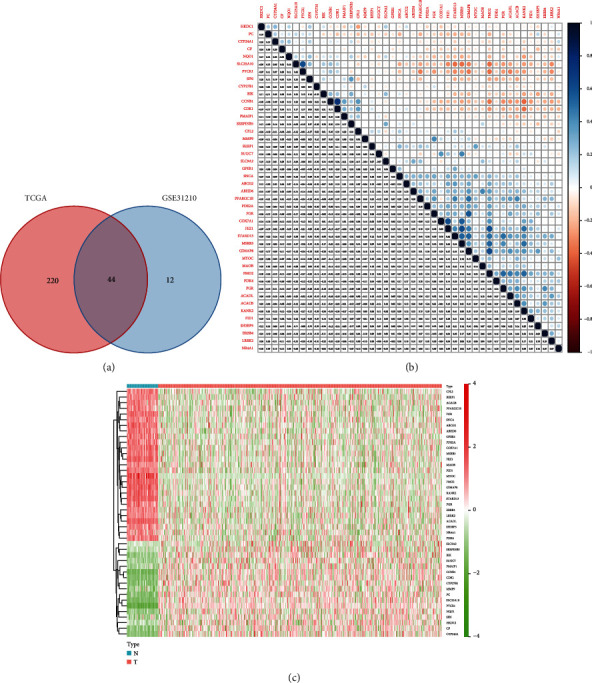
A total of 44 overlapping differentially expressed MTRGs. (a) Venn diagram showing 44 overlapping genes between the two cohorts. (b) Correlogram showing the connection between 44 MTRGs. (c) Heat map showing the expression of 44 MTRGs.

Among the 1513 MTRGs, 264 genes were differentially expressed in TCGA-LUAD cohort and 56 genes were differentially expressed in GSE31210 cohort. There were 44 overlapping differentially expressed MTRGs in two cohorts (Figure 1(a)). Most of these 44 genes are interrelated (Figure 1(b)). The heat map of these 44 genes is shown in Figure 1(c).

### 3.2. Development and Verification of Prediction Signature

**Figure 2:**
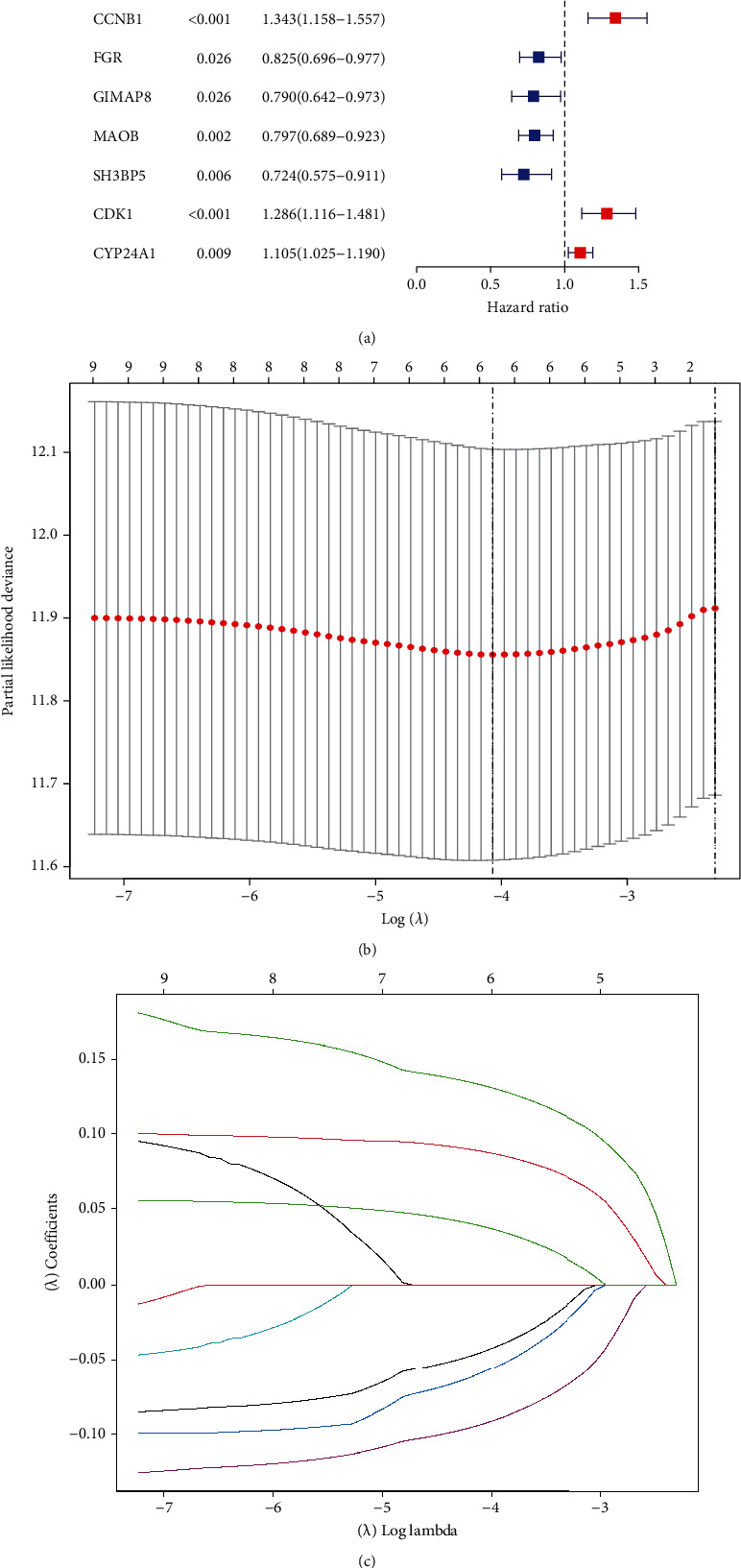
Construction of the signature. (a) Univariate Cox regression analysis screening nine MTRGs associated with prognosis of LUAD. (b) LASSO analysis exploring the minimum lambda value. (c) LASSO detecting six genes for signature construction according to the minimum lambda value.

**Figure 3:**
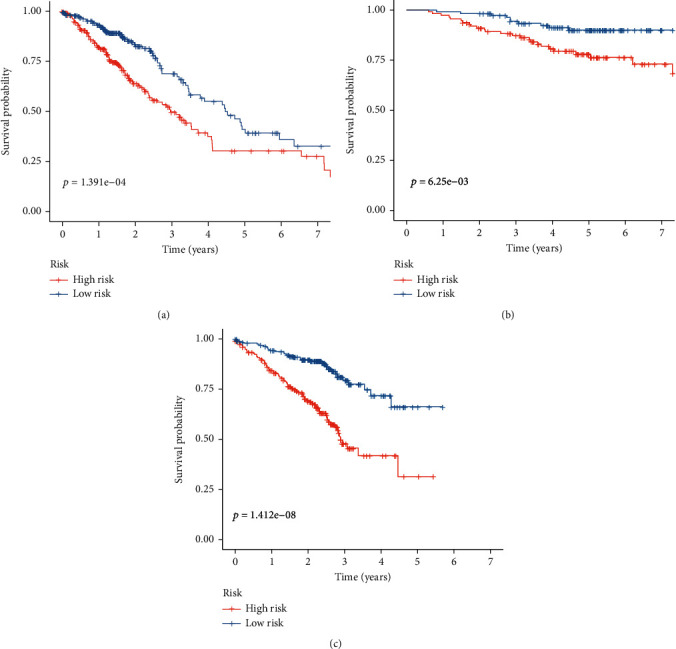
Survival analyses of HR and LR groups. (a) TCGA-LUAD. (b) GSE31210. (c) GSE72094.

**Figure 4:**
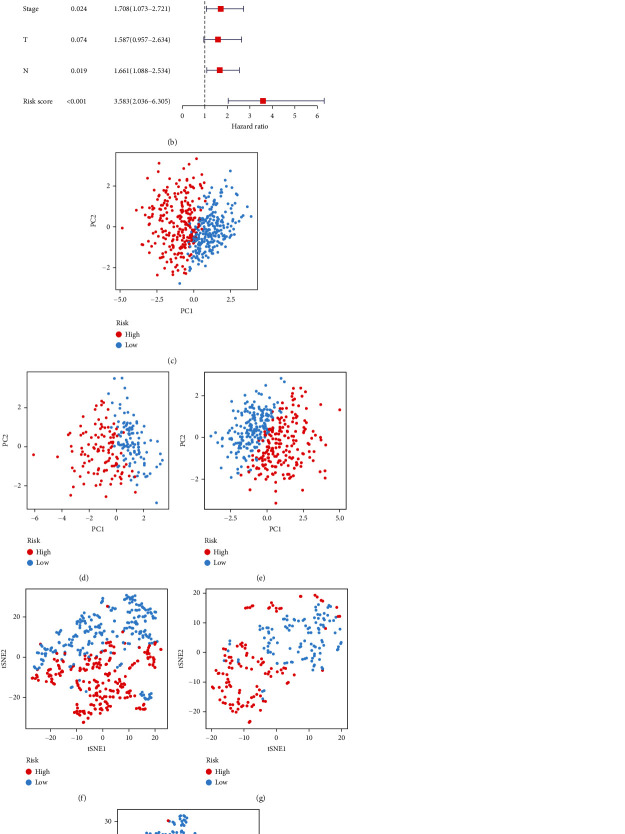
Performance analysis of the signature. (a) Univariate Cox regression analysis showing the independence of the signature. (b) Multivariate Cox regression analysis showing the independence of the signature. (c, f) TCGA-LUAD. (d, g) GSE31210. (e, h) GSE72094. (c–e) PCA analysis. (f–h) tSNE analysis.

**Figure 5:**
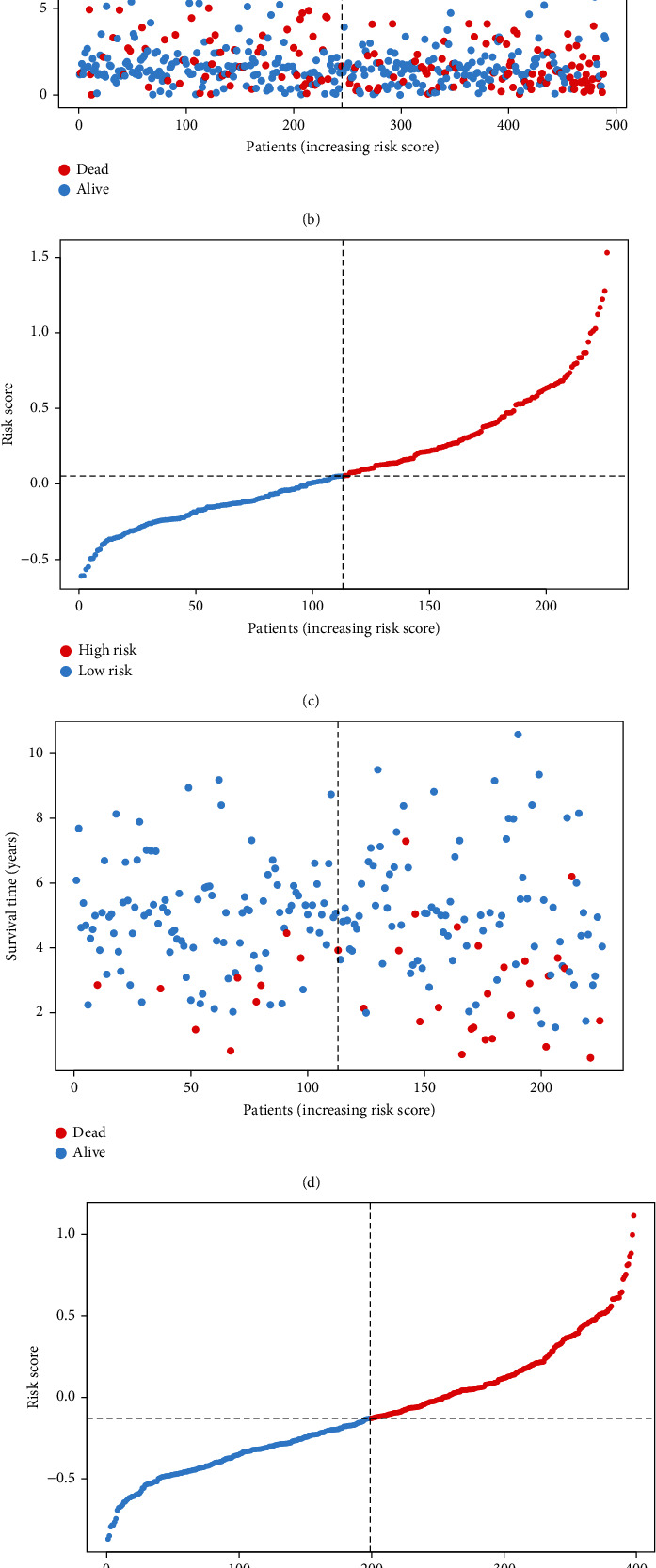
Relationship between risk score and survival status of patients. (a, c, e) Risk distribution curves. (b, d, f) Survival state curves. (a, b) TCGA-LUAD. (c, d) GSE31210. (e, f) GSE72094.

Among the 44 overlapping differentially expressed MTRGs, nine genes were associated with prognosis of LUAD (Figure 2(a)). When the penalty parameter lambda was the minimum, there were six genes meeting the conditions of constructing the signature (Figures 2(b) and 2(c)). The signature was formulated as (0.089163325)∗SERPINB5 + (0.133420615)∗CCNB1 + (−0.058389627)∗FGR + (−0.092777162)∗MAOB + (−0.044191822)∗SH3BP5 + (0.038966882)∗CYP24A1. The survival analysis of TCGA-LUAD and two validation cohorts exhibited that prognosis of patients in the HR group was significantly worse than that in the LR group (Figures 3(a)–3(c)). Cox regression analyses showed that the risk score was an independent predictor of LUAD prognosis (Figures 4(a) and 4(b)). PCA and tSNE analyses demonstrated that the signature can well distinguish between HR and LR patients (Figures 4(c)–4(h)). What is more, the distribution of risk score was visualized as scatterplots (Figures 5(a)–5(f)).

### 3.3. Subgroup Analysis of Clinical Characteristics

**Figure 6:**
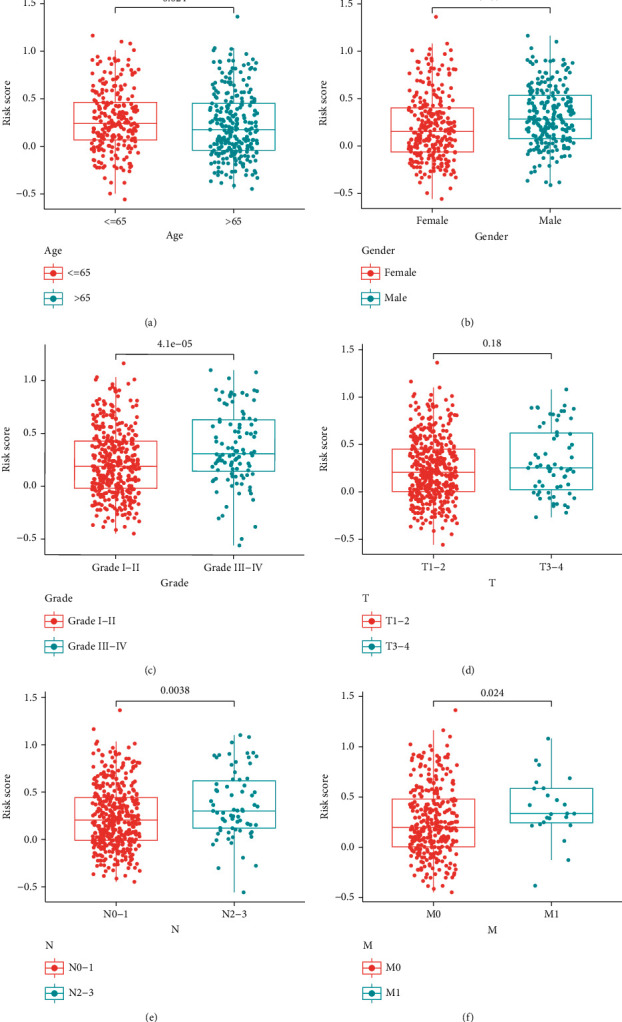
Subgroup analysis of clinical characteristics. (a) Subgroups of age. (b) Subgroups of gender. (c) Subgroups of grade. (d) Subgroups of T stage. (e) Subgroups of N stage. (f) Subgroups of M stage.

In subgroups of available clinical features, the signature showed accurate and stable performance. Except that there was no difference in risk scores between T stage subgroups, there were significant differences in risk scores among other clinical characteristic subgroups. The risk scores of ≤65, female, N2-3, M1, and III-IV groups were higher than those of >65, female, N0-1, M0, and I-II groups, respectively (Figures 6(a)–6(f)).

### 3.4. Immunity Analysis

**Figure 7:**
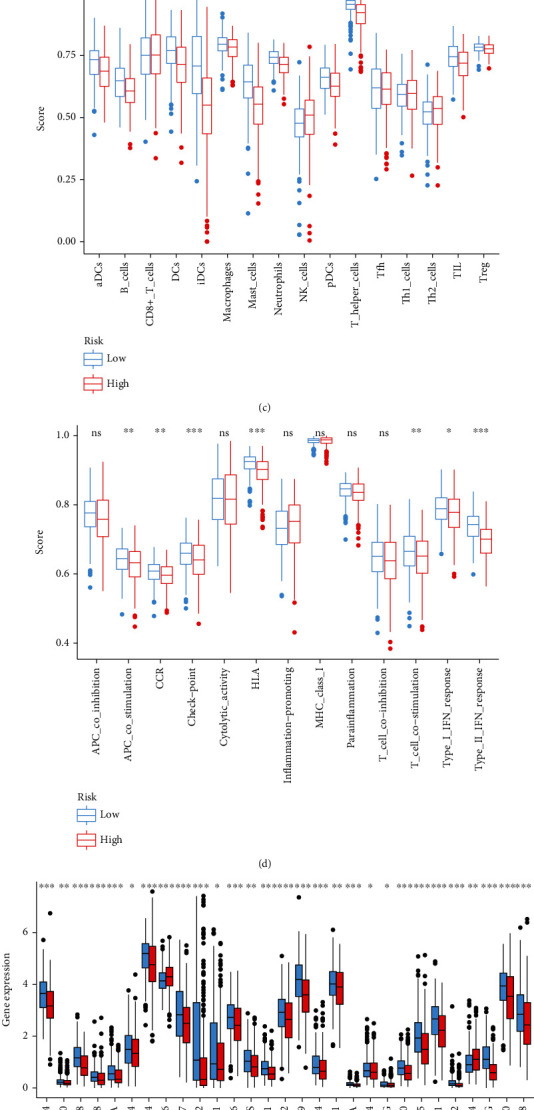
Immunity-related analyses. (a) ESTIMATE score of stromal cells. (b) ESTIMATE score of immune cells. (c) Infiltration score of immune cells. (d) Activity of immunity-related function and pathways. (e) Expression of 29 immune checkpoints. (f) TIDE score. (g) Dysfunction score.

The ESTIMATE scores of stromal and immune cells were negatively correlated with the risk score (Figures 7(a) and 7(b)). As with the results of ESTIMATE score, the degree of immune cell infiltration in the LR group was higher than that in the HR score group, such as TIL, Treg, and B cells (Figure 7(c)). What is more, the immune-related functions and pathways of the LR group were more active than those in the HR group, such as APC costimulation, checkpoint, and T cell costimulation (Figure 7(d)).

The expression level of many immune checkpoints was higher in the LR group than in the HR group, such as CTLA-4, which is an immune checkpoint with more research at present (Figure 7(e)). However, the expression of TNFSF4 was higher in the HR group. However, the TIDE score of the LR group was higher than that of the HR group (Figure 7(f)). The dysfunction score of the LR group was higher than that of the HR group, indicating that the ability of T cells to anticancer cells was weaker in the LR group than in the HR group (Figure 7(g)).

It was found that infiltration of immune cells was related to the expression of signature genes. The expression of CCNB1 was positively correlated with CD8+ T cell and neutrophil and negatively correlated with CD4+ T cell, B cell, macrophage, and dendritic cell. SH3BP5 was positively correlated with macrophage and neutrophil and negatively correlated with other four immune cells. Except neutrophils, CYP24A1 was negatively correlated with five other immune cells. FGR, SH3BP5, and MAOB were positively correlated with six immune cells (Figures S1A–S1F).

### 3.5. Cancer Stem Cell Infiltration Analysis

To explore the association between risk score and cancer stem cell infiltration, we performed cancer stem cell infiltration analysis. The values of *R* were 0.59 (*p* 2.2*e* − 16) and 0.25 (*p* = 2.1*e* − 07) in the RNA score and DNA score, respectively (Figures S2A and S2B).

### 3.6. Tumor Mutation Burden Analysis

There were significant differences in TMB between the two risk groups. The mutation level of the HR group was higher (Figure S2C). It also showed that mutation rate is 94.65% in the HR group and 82.7% in the LR group (Figures S2D and S2E). The most common mutation type and gene in both risk groups were missense mutation and TP53, respectively. The survival analysis showed that the overall survival rate of the H-TMB+low risk group was the highest among the four groups (Figure S2F).

### 3.7. Gene Set Enrichment Analysis

The results of GO showed that the gene set of the HR group was enriched in the cell cycle, such as DNA replication, centromere complex assembly, and G2 phase transition (Figure S2G). The gene set of the LR group was involved in ciliary plasm, cilium movement, axoneme assembly, and so on (Figure S2H). KEGG showed that pathways of the HR group were enriched in cell cycle, DNA replication, ribosome, and so on and pathways of the LR group were enriched in asthma, cell adhesion molecule, hematopoietic cell lineage, and so on (Figures S2I and S2J).

### 3.8. Exploring Sensitive Drugs

According to the *p* value from small to large row, the results of the first 16 analyses are showed in Figure S3, and more results are provided in Table S1. It showed that CYP24A1, FGR, and MAOB were sensitive to nandrolone phenpropionate. CCNB1 was sensitive to 6-thioguanine and allopurinol, while resistant to denileukin diftitox Ontak. While SERPINB5 was sensitive to sunitinib, NMS-E628, and LOXO-101, it was resistant to multiple drugs, such as cisplatin, gemcitabine, and etoposide. SH3BP5 was sensitive to pipamperone, temsirolimus, vemurafenib, and so on.

## 4. Discussion

LUAD is currently being treated with a variety of methods, including surgery, radiation therapy, and chemotherapy [34]. The survival rates of LUAD patients have improved somewhat as treatment strategies for this cancer have been developed [35]. For those who have advanced LUAD, the prognosis remains poor. Patients with LUAD have a 50% lower chance of surviving if they develop metastasis or recurrence, which is the primary cause of the poor prognosis [36, 37]. Nonspecific symptoms of LUAD make it difficult to diagnose the disease, which delays treatment. Therefore, new and effective prognostic indicators are urgently needed in LUAD. The present development of bioinformatics technologies enables powerful, high-throughput methods for screening molecular biomarkers and indications of prognosis in a variety of cancers and other diseases.

For cell growth, differentiation, and apoptosis, mitochondria serve as metabolic hubs that govern the flow of metabolites and energy [38]. Thus, cancer-related biological processes such as tumor initiation, development and invasion, metastasis, and resistance to anticancer treatments are all dependent on mitochondria [39, 40]. A number of recent studies have shown that mitochondrial metabolism could be a viable target for cancer therapy since tumors change a number of mitochondrial metabolic processes [41, 42]. In addition, several prognostic models based on mitochondrial-related genes were identified in some types of tumors, such as bladder cancer and prostate cancer [29, 30]. However, the prognostic model based on mitochondrial-related genes was rarely reported. In this study, we identified 44 overlapping differentially expressed MTRGs in GSE31210 and TCGA datasets. Survival assays revealed that nine genes were associated with prognosis of LUAD among 44 overlapping differentially expressed MTRGs. When the penalty parameter lambda was the minimum, there were six genes meeting the conditions of constructing the signature. Then, we used SERPINB5, CCNB1, FGR, MAOB, SH3BP5, and CYP24A1 to develop a prognostic model. The survival analysis of TCGA-LUAD and two validation cohorts exhibited that prognosis of patients in the HR group was significantly worse than that in the LR group. Our findings highlighted the potential of this novel model used as a novel biomarker for LUAD patients. The function of the above genes in several tumors has been studied. However, their association with LUAD was rarely reported. Thus, more experiments were needed to focus on their tumor-related function in LUAD.

LUAD survival was greatly influenced by the degree of immune infiltration [43]. Immune cells in the tumor microenvironment can be used to predict the prognosis of many malignancies, including cervical cancer and esophageal carcinoma, as well as kidney renal clear cell carcinoma, according to previous researches [44–46]. Immune infiltration ratings were modified by the expression of six genes in this investigation. This finding suggested that the immune system of LUAD is linked to the prognostic value of the risk score. R platform's ESTIMATE method was then used to determine the immune cell subtype. According to our findings, the two groups with differing risk scores displayed distinct immune cell subsets. It has been demonstrated that poor survival and poor prognosis are strongly linked to an imbalance in the immune cell component ratio in cancer patients [47, 48]. According to a prior study, CD8+ T lymphocytes secrete granulocyte and perforin to attack tumor cells [49]. As with the results of ESTIMATE score, the degree of immune cell infiltration in the LR group was higher than that in the HR group, such as TIL, Treg, and B cells. This implies that an imbalance of TIL, Treg, and B cells may influence the survival rate of patients in the high-risk group. Our research also shows that the critical genes were sensitive to several drugs. This indicated that the combination of immunotherapy and chemotherapy can benefit the high-risk group of patients, paving the way for precise and tailored treatment for LUAD sufferers.

TMB is a measure of the number of somatic gene coding mistakes, base substitutions, insertions, and deletions found in one million bases of genomic DNA [50]. The greater the TMB, the more altered the cancer cell is, making it easier for the immune system to identify and kill it [51]. Besides, immune cell infiltration and an inflammatory phenotype are hallmarks of malignancies that react to checkpoint-inhibiting drugs. In this study, the OS of the high-TMB+HR group was better than that of the low-TMB+HR group, and the OS of the high-TMB+LR group was better than that of the low-TMB+LR group, suggesting that higher TMB is beneficial to OS of LUAD patients. In addition to the ability to self-renew and differentiate through symmetrical or asymmetrical cell division, CSCs are endowed with stem cell traits. Radiation and chemotherapy are not able to kill CSCs because of their ability to self-renew and generate progenitor cells. The infiltration of cancer stem cells in the HR group was higher than that in the LR group, indicating that LUAD cells in the HR group had more obvious stem cell characteristics and lower degree of cell differentiation. These may also explain the lower OS in the HR group.

In spite of the above studies, our prognostic model still faced some limitations in terms of clinical application. There is still a need for future studies to verify that the high-throughput data accumulated from a large number of samples has been optimally applied. More studies are needed to determine the specific roles of the six LUAD genes in in vitro and in vivo experiments.

## 5. Conclusion

According to our analysis of TCGA and GEO LUAD cohorts, a prognostic MTRG signature was discovered. The prognosis could be predicted using this gene signature alone. The correlations between our signature and immunotherapy-related biomarkers suggest that our signature can be used to predict the effectiveness of immunotherapy. In the future, individualized treatments and medical decision-making may benefit from using the predicted model.

## Data Availability

The data used to support the findings of this study are available from the corresponding author upon request.

## Supplementary Materials

Supplementary Materials Figure S1: correlation between signature genes and immune cell infiltration. (A) CCNB1. (B) CYP24A1. (C) FGR. (D) MAOB. (E) SERPINB5. (F) SH3BP5. Figure S2: cancer stem cell infiltration, TMB, and GSEA analyses. (A) Evaluation of infiltration of cancer stem cells at RNA level. (B) Evaluation of infiltration of cancer stem cells at the DNA level. (C) TMB. (D) Mutation of the HR group. (E) Mutation of the LR group. (F) Survival analysis of four groups. (G) GO analysis of the HR group. (H) GO analysis of the LR group. (I) KEGG analysis of the HR group. (J) KEGG analysis of the LR group. Figure S3: drugs. Table S1: drugs.
