# Supplementary material for: A Novel Mitochondrial-Related Gene Signature for the Tumor Immune Microenvironment Evaluation and Prognosis Prediction in Lung Adenocarcinoma
Source: J Immunol Res. 2022 May 25;2022:5366185. doi: 10.1155/2022/5366185 (PMC9159837; doi:10.1155/2022/5366185)
Supplement: Supplementary Materials — Figure S1: correlation between signature genes and immune cell infiltration. (A) CCNB1. (B) CYP24A1. (C) FGR. (D) MAOB. (E) SERPINB5. (F) SH3BP5. Figure S2: cancer stem cell infiltration, TMB, and GSEA analyses. (A) Evaluation of infiltration of cancer stem cells at RNA level. (B) Evaluation of infiltration of cancer stem cells at the DNA level. (C) TMB. (D) Mutation of the HR group. (E) Mutation of the LR group. (F) Survival analysis of four groups. (G) GO analysis of the HR group. (H) GO analysis of the LR group. (I) KEGG analysis of the HR group. (J) KEGG analysis of the LR group. Figure S3: drugs. Table S1: drugs. [file 5366185.f1.zip › 5366185.f1/Figure S3.pdf]

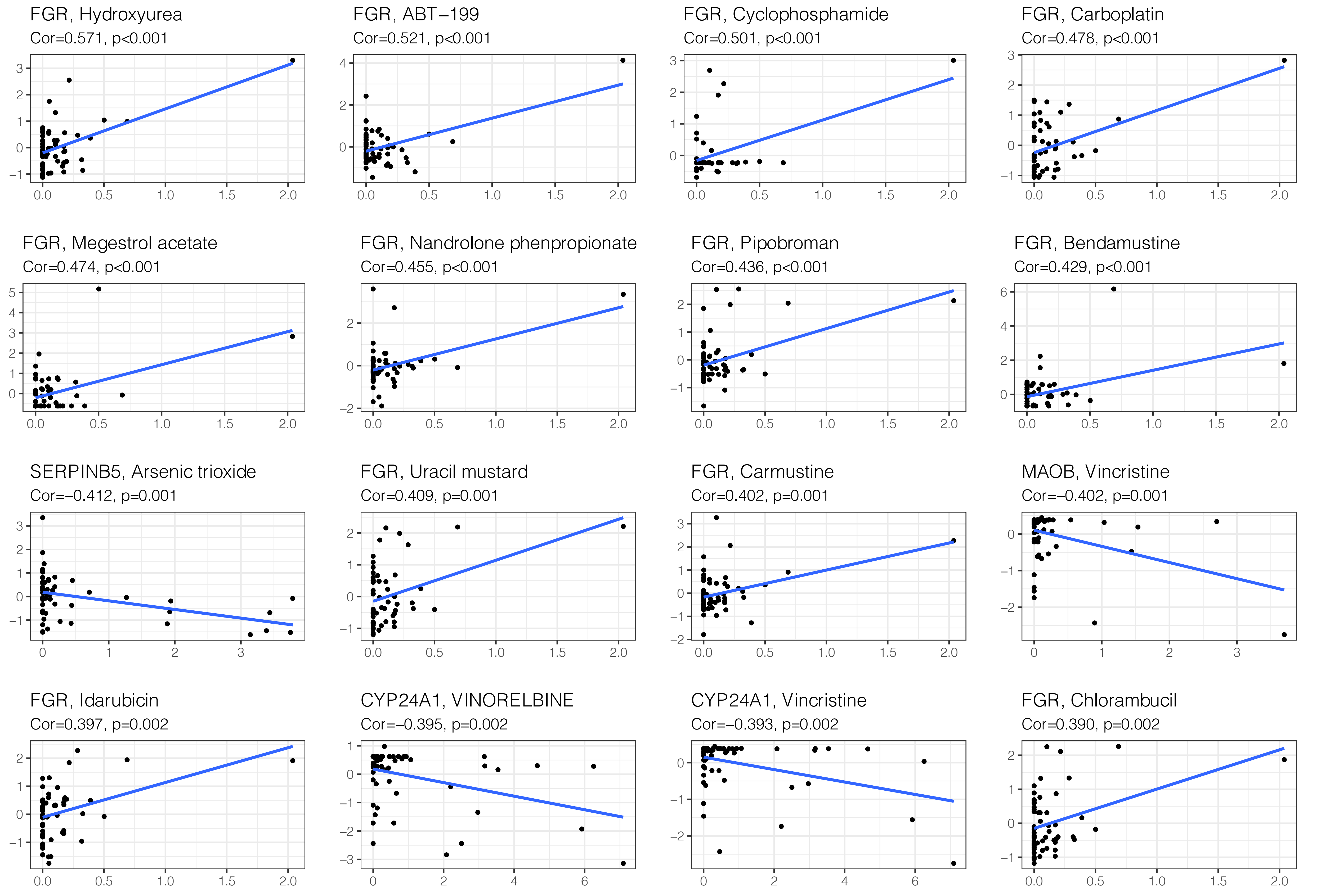
FGR, Hydroxyurea
Cor=0.571, p<0.001
FGR, ABT−199
Cor=0.521, p<0.001
FGR, Cyclophosphamide
Cor=0.501, p<0.001
FGR, Carboplatin
Cor=0.478, p<0.001
FGR, Megestrol acetate
Cor=0.474, p<0.001
FGR, Nandrolone phenpropionate
Cor=0.455, p<0.001
FGR, Pipobroman
Cor=0.436, p<0.001
FGR, Bendamustine
Cor=0.429, p<0.001
SERPINB5, Arsenic trioxide
Cor=−0.412, p=0.001
FGR, Uracil mustard
Cor=0.409, p=0.001
FGR, Carmustine
Cor=0.402, p=0.001
MAOB, Vincristine
Cor=−0.402, p=0.001
FGR, Idarubicin
Cor=0.397, p=0.002
CYP24A1, VINORELBINE
Cor=−0.395, p=0.002
CYP24A1, Vincristine
Cor=−0.393, p=0.002
FGR, Chlorambucil
Cor=0.390, p=0.002
