# Supplementary material for: A Novel Mitochondrial-Related Gene Signature for the Tumor Immune Microenvironment Evaluation and Prognosis Prediction in Lung Adenocarcinoma
Source: J Immunol Res. 2022 May 25;2022:5366185. doi: 10.1155/2022/5366185 (PMC9159837; doi:10.1155/2022/5366185)
Supplement: Supplementary Materials — Figure S1: correlation between signature genes and immune cell infiltration. (A) CCNB1. (B) CYP24A1. (C) FGR. (D) MAOB. (E) SERPINB5. (F) SH3BP5. Figure S2: cancer stem cell infiltration, TMB, and GSEA analyses. (A) Evaluation of infiltration of cancer stem cells at RNA level. (B) Evaluation of infiltration of cancer stem cells at the DNA level. (C) TMB. (D) Mutation of the HR group. (E) Mutation of the LR group. (F) Survival analysis of four groups. (G) GO analysis of the HR group. (H) GO analysis of the LR group. (I) KEGG analysis of the HR group. (J) KEGG analysis of the LR group. Figure S3: drugs. Table S1: drugs. [file 5366185.f1.zip › 5366185.f1/Table S1.docx]

Table S1 Drug.

| Gene | Drug | cor | pvalue |
| --- | --- | --- | --- |
| CCNB1 | Denileukin Diftitox Ontak | -0.35347 | 0.005598 |
| CCNB1 | 6-Thioguanine | 0.340144 | 0.007835 |
| CCNB1 | Allopurinol | 0.268667 | 0.037927 |
| CYP24A1 | VINORELBINE | -0.39451 | 0.001814 |
| CYP24A1 | Vincristine | -0.39341 | 0.001873 |
| CYP24A1 | Gefitinib | 0.378024 | 0.002902 |
| CYP24A1 | umbralisib | 0.347087 | 0.006588 |
| CYP24A1 | Erlotinib | 0.34627 | 0.006725 |
| CYP24A1 | Neratinib | 0.342981 | 0.007302 |
| CYP24A1 | Vinblastine | -0.3145 | 0.014391 |
| CYP24A1 | Vandetanib | 0.311798 | 0.015302 |
| CYP24A1 | Afatinib | 0.291661 | 0.023755 |
| CYP24A1 | 6-MERCAPTOPURINE | -0.28742 | 0.025964 |
| CYP24A1 | Dacomitinib | 0.283521 | 0.028149 |
| CYP24A1 | Nandrolone phenpropionate | 0.267791 | 0.038582 |
| CYP24A1 | Pipamperone | -0.26437 | 0.04123 |
| FGR | Hydroxyurea | 0.571114 | 1.89E-06 |
| FGR | ABT-199 | 0.520935 | 1.98E-05 |
| FGR | Cyclophosphamide | 0.501094 | 4.54E-05 |
| FGR | Carboplatin | 0.477571 | 0.000114 |
| FGR | Megestrol acetate | 0.474339 | 0.000129 |
| FGR | Nandrolone phenpropionate | 0.45531 | 0.000257 |
| FGR | Pipobroman | 0.43593 | 0.000498 |
| FGR | Bendamustine | 0.42873 | 0.000631 |
| FGR | Uracil mustard | 0.408963 | 0.001177 |
| FGR | Carmustine | 0.402215 | 0.001444 |
| FGR | Idarubicin | 0.397401 | 0.001666 |
| FGR | Chlorambucil | 0.39041 | 0.002043 |
| FGR | Thiotepa | 0.387405 | 0.002228 |
| FGR | Etoposide | 0.386865 | 0.002262 |
| FGR | Melphalan | 0.383214 | 0.002509 |
| FGR | Triethylenemelamine | 0.369257 | 0.003691 |
| FGR | Isotretinoin | 0.355854 | 0.005264 |
| FGR | Cisplatin | 0.339799 | 0.007901 |
| FGR | Lomustine | 0.334761 | 0.008938 |
| FGR | Teniposide | 0.327549 | 0.010628 |
| FGR | Valrubicin | 0.309278 | 0.016193 |
| FGR | Nelfinavir | 0.302985 | 0.018615 |
| FGR | Dasatinib | -0.29721 | 0.021101 |
| FGR | Nitrogen mustard | 0.294866 | 0.022191 |
| FGR | Asparaginase | 0.292308 | 0.023431 |
| FGR | DIGOXIN | 0.292102 | 0.023534 |
| FGR | Cladribine | 0.287663 | 0.025835 |
| FGR | Daunorubicin | 0.286514 | 0.02646 |
| FGR | Ifosfamide | 0.28635 | 0.02655 |
| FGR | Parthenolide | 0.283186 | 0.028343 |
| FGR | Palbociclib | 0.281558 | 0.029305 |
| FGR | DACARBAZINE | 0.276883 | 0.03222 |
| FGR | Epirubicin | 0.271037 | 0.036201 |
| FGR | Dexrazoxane | 0.269098 | 0.037608 |
| FGR | Oxaliplatin | 0.266831 | 0.039311 |
| FGR | Dromostanolone Propionate | 0.263061 | 0.042284 |
| MAOB | Vincristine | -0.40156 | 0.001473 |
| MAOB | Megestrol acetate | 0.349238 | 0.006238 |
| MAOB | Gefitinib | 0.303168 | 0.018541 |
| MAOB | 6-MERCAPTOPURINE | -0.30228 | 0.018903 |
| MAOB | Vandetanib | 0.297199 | 0.021108 |
| MAOB | Floxuridine | -0.29533 | 0.021974 |
| MAOB | AZACITIDINE | -0.29157 | 0.023798 |
| MAOB | Abiraterone | 0.287477 | 0.025935 |
| MAOB | Simvastatin | 0.280011 | 0.030244 |
| MAOB | Nandrolone phenpropionate | 0.27951 | 0.030553 |
| MAOB | VINORELBINE | -0.26166 | 0.043432 |
| MAOB | Afatinib | 0.258938 | 0.045744 |
| MAOB | Fluorouracil | -0.25867 | 0.045976 |
| SERPINB5 | Arsenic trioxide | -0.41184 | 0.001078 |
| SERPINB5 | LOXO-101 | 0.381401 | 0.002641 |
| SERPINB5 | Ixazomib citrate | -0.36166 | 0.004521 |
| SERPINB5 | ARSENIC TRIOXIDE | -0.33897 | 0.008064 |
| SERPINB5 | Cisplatin | -0.33618 | 0.008635 |
| SERPINB5 | Gemcitabine | -0.31404 | 0.014544 |
| SERPINB5 | Carboplatin | -0.30676 | 0.017129 |
| SERPINB5 | Uracil mustard | -0.30158 | 0.019197 |
| SERPINB5 | tfdu | -0.29961 | 0.020039 |
| SERPINB5 | Bortezomib | -0.29823 | 0.020643 |
| SERPINB5 | Carmustine | -0.29262 | 0.023275 |
| SERPINB5 | Chlorambucil | -0.28565 | 0.026941 |
| SERPINB5 | NMS-E628 | 0.283758 | 0.028012 |
| SERPINB5 | Melphalan | -0.28344 | 0.028198 |
| SERPINB5 | Ifosfamide | -0.28251 | 0.02874 |
| SERPINB5 | Etoposide | -0.27659 | 0.032409 |
| SERPINB5 | Temsirolimus | -0.27543 | 0.033175 |
| SERPINB5 | RAPAMYCIN | -0.27071 | 0.036432 |
| SERPINB5 | Pazopanib | -0.26557 | 0.040289 |
| SERPINB5 | Sunitinib | 0.264057 | 0.041481 |
| SERPINB5 | Topotecan | -0.25836 | 0.04625 |
| SERPINB5 | Bleomycin | -0.25618 | 0.048182 |
| SERPINB5 | MITOXANTRONE | -0.25606 | 0.048297 |
| SERPINB5 | Everolimus | -0.25578 | 0.048546 |
| SH3BP5 | Nelfinavir | 0.345989 | 0.006773 |
| SH3BP5 | Dasatinib | -0.31394 | 0.014577 |
| SH3BP5 | Vemurafenib | 0.308959 | 0.016309 |
| SH3BP5 | Dabrafenib | 0.296307 | 0.021517 |
| SH3BP5 | Temsirolimus | 0.294689 | 0.022275 |
| SH3BP5 | ETHINYL ESTRADIOL | 0.292795 | 0.023191 |
| SH3BP5 | Estramustine | 0.281614 | 0.029271 |
| SH3BP5 | Denileukin Diftitox Ontak | 0.277518 | 0.03181 |
| SH3BP5 | Dacomitinib | -0.26514 | 0.040621 |
| SH3BP5 | RAPAMYCIN | 0.260833 | 0.044126 |
| SH3BP5 | Pipamperone | 0.258362 | 0.046246 |
